# Degradation potential of alkanes by diverse oil-degrading bacteria from deep-sea sediments of Haima cold seep areas, South China Sea

**DOI:** 10.3389/fmicb.2022.920067

**Published:** 2022-10-19

**Authors:** Lina Lyu, Jie Li, Yu Chen, Zhimao Mai, Lin Wang, Qiqi Li, Si Zhang

**Affiliations:** ^1^CAS Key Laboratory of Tropical Marine Bio-resources and Ecology, South China Sea Institute of Oceanology, Chinese Academy of Sciences, Guangzhou, China; ^2^Southern Marine Science and Engineering Guangdong Laboratory (Guangzhou), Guangzhou, China

**Keywords:** oil, alkane, degrading bacteria, biodiversity, Haima cold seep

## Abstract

Marine oil spills are a significant concern worldwide, destroying the ecological environment and threatening the survival of marine life. Various oil-degrading bacteria have been widely reported in marine environments in response to marine oil pollution. However, little information is known about culturable oil-degrading bacteria in cold seep of the deep-sea environments, which are rich in hydrocarbons. This study enriched five oil-degrading consortia from sediments collected from the Haima cold seep areas of the South China Sea. *Parvibaculum*, *Erythrobacter*, *Acinetobacter*, *Alcanivorax*, *Pseudomonas*, *Marinobacter*, *Halomonas*, and *Idiomarina* were the dominant genera. Further results of bacterial growth and degradation ability tests indicated seven efficient alkane-degrading bacteria belonging to *Acinetobacter*, *Alcanivorax*, *Kangiella*, *Limimaricola*, *Marinobacter, Flavobacterium*, and *Paracoccus*, whose degradation rates were higher in crude oil (70.3–78.0%) than that in diesel oil (62.7–66.3%). From the view of carbon chain length, alkane degradation rates were medium chains > long chains > short chains. In addition, *Kangiella aquimarina* F7, *Acinetobacter venetianus* F1, *Limimaricola variabilis* F8, *Marinobacter nauticus* J5, *Flavobacterium sediminis* N3, and *Paracoccus sediminilitoris* N6 were first identified as oil-degrading bacteria from deep-sea environments. This study will provide insight into the bacterial community structures and oil-degrading bacterial diversity in the Haima cold seep areas, South China Sea, and offer bacterial resources to oil bioremediation applications.

## Introduction

Petroleum hydrocarbons have been common organic pollutants in marine environments for millions of years (Hassanshahian et al., 2014; Hazen et al., 2016; Akyuz and Celik, 2018; Paniagua-Michel and Fathepure, 2018; Love et al., 2021). With petroleum transportation industry development and exploitation in offshore sea areas, the occurrence frequency of marine oil spills has shown increasing trends over time (Ma et al., 2021b). It was estimated that approximately 1.3 million tons of petroleum hydrocarbons enter the marine environment annually from anthropogenic and natural sources (Hassanshahian et al., 2012; Ventikos and Sotiropoulos, 2014; Hazen et al., 2016). Among them, approximately 0.6 million tons of petroleum hydrocarbons were derived from natural seeps and could cover all oceans in the world with a thickness of 20 molecules (Head et al., 2006; Gao et al., 2015). Thus, petroleum hydrocarbon pollution poses a significant threat to marine ecosystems (Head et al., 2006; Emtiazi et al., 2009; Hazen et al., 2010; Hassanshahian et al., 2014; Jagtap et al., 2021). Therefore, it is necessary to develop eco-friendly technologies to remove oil contamination from marine environments.

Previous studies have widely reported many physical, chemistry, and bioremediation technologies. Compared to physical and chemical methods, microbial remediation has more advantages of low cost, high efficiency, and sustainability (Kujawinski et al., 2011; Paniagua-Michel and Fathepure, 2018; Zhao et al., 2018; Poddar et al., 2019; Socolofsky et al., 2019). Bacteria are better oil degraders than other microorganisms (Das and Chandran, 2011; Shi et al., 2021). Hence, it is critical to obtain highly effective oil-degrading bacteria. More than 70 genera of marine bacteria have been successfully isolated and identified as oil degraders (Bao et al., 2012; Ferrari et al., 2019). Some of them were obtained from deep-sea environments with unique habitats. For example, Ma et al. (2021b) obtained 35 oil-degrading bacteria from sediments in the deep-sea hydrothermal areas of the South Mid-Atlantic Ridge. Thirty-four PAH-degrading isolates were obtained from the deep-sea water column of the SWIR at a depth of 4,766 m (Shao et al., 2015). Gao et al. (2015) isolated 11 strains of oil-degrading bacteria from the deep-sea sediments of the South Mid-Atlantic Ridge. Shi et al. (2021) isolated 162 strains of oil-degrading bacteria from the Southwest Mid-Indian Ocean Ridge sediments.

Cold seeps are an extreme environment of low temperatures, high hydrostatic pressure, and the absence of light in the deep sea and have regular influxes of petroleum hydrocarbons due to natural seepage (Niu et al., 2017; Potts et al., 2018; Cui et al., 2019; Van Landuyt et al., 2020). Hydrocarbons, as carbon sources, can promote the growth of oil-degrading bacteria in cold seep ecosystems (Pachiadaki and Kormas, 2013; Cui et al., 2019). Consequently, it is scientific to screen oil-degrading bacteria from deep-sea cold seep environments. Chemical compositions and geographic locations vary in different cold seep areas, which may harbor distinct microbial populations (Pop Ristova et al., 2012; Ruff et al., 2015; Zhang et al., 2020). Thus, the diversity of oil-degrading bacteria may vary in different cold seep areas. The Haima cold seep, a newly discovered cold seep, was first reported on the northwestern slope of the SCS in 2015 (Liang et al., 2017). To date, minimal studies have been conducted on the microbial communities of Haima cold seeps (Niu et al., 2017; Zhuang et al., 2019; Jing et al., 2020; Ling et al., 2020). The diversity of oil-degrading bacteria in the Haima cold seep areas remains unexplored.

In the present study, sediments were collected from the Haima cold seep areas of the South China Sea. Oil-degrading consortia were enriched from the sediments with crude and diesel oil as the sole carbon and energy sources. This research investigated the effects of oils on microbial community, studied oil-degrading bacterial diversity, screened high-efficiency oil-degrading strains, and explored the degradation ability of alkanes by high-efficiency oil-degrading bacteria. This study will provide a new perspective for understanding the community structure and biodiversity of culturable oil-degrading bacteria in the deep-sea sediments of the Haima cold seep. In addition, this study will provide bacterial resources for oil bioremediation applications.

## Materials and methods

### Sediment collection

The five surface sediments were collected from different stations of the Haima cold seep area (16.9°N, 110.4°E) of the northern South China Sea using the *Haima* ROV during the cruise R/V *Haiyang VI* of the Guangzhou Marine Geological Survey, China, in September 2020. Five sediment samples were recorded as F, J, I, N, and G. Sediments of F and J were collected from the different degrees of the active cold seep areas, which are covered by a mass of mussels and contain abundant methane gas. The sediment of I was obtained from the non-active cold seep area without organisms. Sediments of N and G were gained from the inactive cold seep area with clams and the extinct cold seep area with dead mussels, respectively. After collection, the sediment samples were put in sterile centrifuge tubes and immediately stored at 4°C until the experiment started in the laboratory.

### Media and chemicals

In this study, crude oil was obtained from a Shengli Oil Production Plant, China, and diesel oil is marine diesel oil. Marine mineral culture (MMC) medium was used to enrich oil-degrading consortia and degradation ability tests for alkanes by oil-degrading bacteria. MMC medium was prepared following descriptions in a paper by Liu and Shao (2005). Specifically, the MMC medium contained three solutions of solution I (1 L), solution II (10 mL), and solution III (10 mL). The solution I was composed of NaCl (24 g), NH_4_NO_3_ (1 g), KCl (0.7 g), KH_2_PO_4_ (2 g), Na_2_HPO_4_ (3 g), and 1 L DDW. Then, the pH was adjusted to 7.4 by using a NaOH solution (10 mol/L). Solution II was only composed of MgSO_4_⋅7H_2_O (35 g) in 100 mL of DDW. Solution III contained CaCl_2_ (2 mg), FeCl_3_⋅6H_2_O (50 mg), CuSO*4* (0.5 mg), MnCl_2_⋅4H_2_O (0.5 mg), and ZnSO_4_⋅7H_2_O (10 mg) in 1 L DDW. Marine Broth 2216 agar (MA, BD Difco) plates were used to isolate strains from oil-degrading consortia. Before use, solution III was sterilized by filtering, and all other media were autoclaved at 121°C for 20 min. For analysis of alkanes, chromatographic grade hexane and anhydrous sodium sulfate were purchased from Tedia (USA) and Sinopharm (Shanghai, China), respectively. All experiments used oil as the sole carbon and energy source.

### Enrichment of oil-degrading consortia

About 1 g of surface sediment for each sample was inoculated into a 100 mL MMC medium containing a 1 g/L mixture of crude and diesel oil (ratio of the concentration of crude oil to diesel oil = 1:1) as the sole carbon and energy sources in a 250 mL Erlenmeyer flask. For the first enrichment, cultures were aerobically incubated at 28°C and 150 rpm in the dark for 7 days. Then, 5 mL of enriched cultures was transferred into 100 mL fresh MMC media with 1 g/L oil and further cultivated for the second enrichment under the same conditions. Similarly, the third enrichment was conducted. After the three continuous enrichments, five different oil-degrading consortia were obtained for further isolation of oil-degrading bacteria.

### Analysis of bacterial community structure

The bacterial community structure of cultures at the beginning of incubation and after every enrichment was analyzed by high-throughput sequencing technology. The total DNA of cultures was extracted using a DNeasy PowerSoil Kit (supplied by QIAGEN GmbH, Germany) according to the protocols. Regions (V3–V4) of the 16S rRNA genes were PCR-amplified with primers 338 F (ACTCCTACGGGAGGCAGCAG) and 806R (GGACTACHVGGGTWTCTAAT) (Shi et al., 2021). After purification, PCR products were sequenced using the Illumina MiSeq sequencing platform (Majorbio, Shanghai, China).

### Isolation, identification, and phylogenetic analysis of oil-degrading bacteria

Bacterial isolation was performed by serial dilution and plating on 1.5% MA plates using 100 μl of culture from each oil-degrading consortium. These plates were incubated at 28°C for 5–7 days. Colonies with distinct morphologies were streaked on fresh MA plates for purification. All obtained bacterial strains were stored in 25% glycerol at –80°C for further analysis. Following the manufacturer’s instructions, the DNA of bacterial strains was extracted by a bacterial genomic DNA extraction kit (Shanghai, SBS Genetech Co., Ltd., China). The 16S rRNA gene sequence was PCR-amplified with primers 27F and 1492R. The PCR products were sequenced by TsingKe Biological Technology Co., Ltd. (Guangzhou, China), using the sanger sequencing platform. Bacterial identification was performed by aligning the 16S rRNA gene sequence in the EzBioCloud database. Based on the 16S rRNA gene sequences of bacterial strains and their closest species, a neighbor-joining phylogenetic tree was constructed by MEGA version 7.0 with 1,000 bootstrap values (Kumar et al., 2007).

### Screening high-efficiency oil-degrading strains

The growth of all isolates was tested in the MMC medium with 1 g/L oil (ratio of crude oil to diesel = 1:1) as the sole carbon and energy source. Specifically, strains were grown on MA plates for 2 days, and fresh colonies were harvested by centrifugation at 5,000 rpm for 10 min. Then, pellets were washed twice with fresh MMC medium and resuspended in fresh MMC medium to make final OD_600_ values of about 1.0. Next, 1 mL culture was inoculated into 100 mL MMC medium supplemented with 1 g/L oil in 250 mL Erlenmeyer flasks and incubated for 11 days at 28°C and 150 rpm in the dark. During incubation, cell growth was measured by monitoring cell turbidity as indicated by optical density at 600 nm (OD_600_) at intervals of 1 day. After incubation, all tested strains were generally divided into three categories based on the OD_600_ values and visual phenomena: (i) small black particles were produced, and bacterial cultures turned to be turbid with the increase in OD_600_ values (Supplementary Figure 1A), (ii) almost no particles were observed, and bacterial cultures turned to be brown with increasing OD_600_ values (Supplementary Figure 1B), (iii) oils were adsorbed on the bottle wall, and no turbidity change was observed in bacterial cultures (Supplementary Figure 1C). From the strains in phenomena (i) and (ii), seven strains with relatively higher OD600 values belonging to different genera were considered high-efficiency oil-degrading strains and used for further characterization of alkane degradation ability.

### Alkane degradation ability by high-efficiency oil-degrading strains

Alkane degradation ability by the selected bacterial strains was studied in the MMC medium with crude or diesel oil as the sole substrate. Briefly, MMC was prepared and divided into 40 mL glass bottles. Each bottle contained 10 mL MMC and 0.1 g crude or diesel oil. Isolates were cultured in MB medium for 2 days, and cells were harvested by centrifugation at 5,000 rpm for 10 min. Pellets were washed twice with fresh MMC medium and resuspended in fresh MMC medium. Next, they were transferred into 40 mL glass bottles to make cultures with final OD_600_ values of about 1.2. Control groups were set as 10 mL uninoculated MMC medium amended with 0.1 g crude or diesel oil. Experiment and control groups were performed in triplicate and incubated at 28°C and 150 rpm in the dark for 20 days. The whole bottle of bacterial cultures before and after 20 days of incubation was stored at –20°C and used to analyze alkanes. The degradation rates of alkanes (D, %) were calculated by subtracting the decreased amounts of alkanes in uninoculated control groups based on the following equation: *D* = [(M1-M2)-M3]/M1 × 100%, where M1 is the weight of alkanes before incubation; M2 is the weight of alkanes after incubation; and M3 is the weight loss of alkanes in control groups.

### Analysis of alkanes in crude and diesel oil by gas chromatography-mass spectrometry

The culture was added and extracted with 10 mL hexane. The sample was then transferred to a centrifuge tube and centrifuged at 2,800 r/min for 10 min. After removing the organic phase in the bottom layer, the water phase in the top layer was extracted for the second time. The third extraction was conducted similarly, and three extraction liquors were combined. Next, 200 μL of the extraction liquor was eluted by 25 mL of hexane in a silica gel column containing the activated silica gel (5 g) and 1.0-cm-high anhydrous granular sodium sulfate. The eluent was concentrated to 1.0 mL under a stream of nitrogen and used for analysis by a 6,890 gas chromatography-5,973 mass selective detector (GC–MS, Agilent, Palo Alto, CA, USA) system equipped with a fused silica capillary HP-5MS column (30 m × 0.25 mm i.d., 0.25 μm film thickness, Agilent, USA).

## Results and discussion

### Effects of oils on bacterial community structures

**FIGURE 1:**
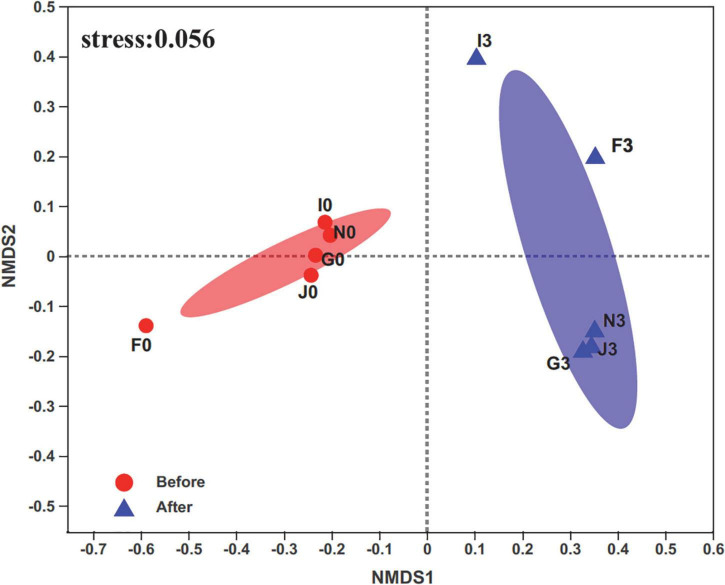
Non-metric multidimensional scaling (NMDS) analysis on the OTU level compares bacterial communities’ clustering patterns in cultures before and after enrichments. F, J, I, N, and G represent five different cultures derived from five surface sediments. Number 0 represents cultures before enrichments; number 3 represents cultures after enrichments.

A total of 829,316 high-quality sequences were retrieved from sequenced samples. The valid sequence numbers were normalized to the minimum reads of 23,037 for analyzing bacterial community diversity and abundance. Then, these sequences were clustered into 2,632 OTUs at 97% nucleotide similarity. Rarefaction curves of the Sobs and Shannon indices at the OTU level showed that a sequencing depth of 23,037 was sufficient for subsequent analysis of bacterial community structures (Supplementary Figure 2). The Wilcoxon rank-sum test for the Chao, Shannon, and Simpson indices revealed significant differences (*p* < 0.05) in the bacterial community structures of cultures before enrichment and after the third enrichment (Supplementary Figure 3). Furthermore, compositions of bacterial communities were different between the two groups of cultures before and after the third enrichment, as revealed by non-metric multidimensional scaling (NMDS) (Figure 1). In addition, the bacterial diversity of the five cultures exhibited an apparent decrease from before enrichment to after the third enrichment because of the selection process of oils (Supplementary Figure 4). These results supported that bacterial community structures were simplified under the effects of oils, such as crude oil, diesel oil, and polycyclic aromatic hydrocarbons (Liu et al., 2011; Sutton et al., 2013; Militon et al., 2015; Meng et al., 2016; Mahjoubi et al., 2021). Oil contamination significantly caused the loss of bacterial diversity in marine environments (Head et al., 2006; Catania et al., 2018; Oyetibo et al., 2021).

**FIGURE 2:**
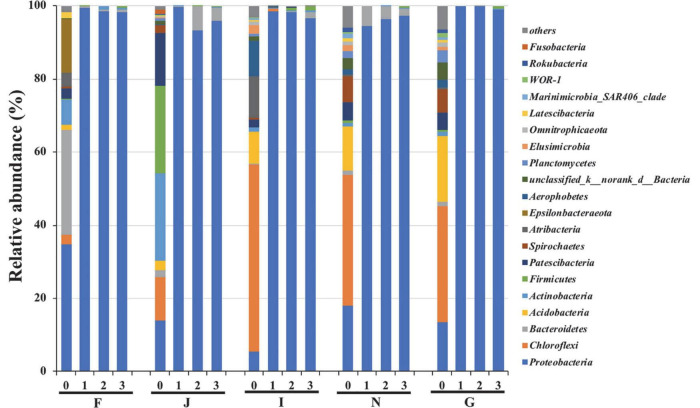
Variations in bacterial community compositions at phylum level during enrichments. 0, 1, 2, and 3 represent cultures before, after the first, after the second, and after the third enrichment. F, J, I, N, and G refer to five different cultures derived from five surface sediments.

The changes in bacterial community compositions during the enrichments are shown in Figure 2. At the phylum level, bacterial communities of the five cultures at the initial phase of enrichment (stage 0) were different. In culture F, the dominant bacteria were Proteobacteria, Bacteroidetes, and Epsilonbacteraeota, with a relative abundance of 34.7, 28.5, and 14.5%, respectively. In culture J, more diverse bacteria of Proteobacteria, Chloroflexi, Actinobacteria, Firmicutes, and Patescibacteria were the dominant bacteria, and their abundances were 14.1, 11.7, 24.0, 23.8, and 14.4%, respectively. The culture I contained the dominant species of Chloroflexi and Atribacteria, with respective abundances of 51.2 and 11.3%, respectively. In cultures N and G, Proteobacteria, Chloroflexi, and Acidobacteria were commonly predominant bacteria, and they had relative abundances of 18.0, 35.8, and 12.1% and 13.6, 31.6, and 17.9%, respectively. After three enrichments, Proteobacteria increased to be the predominant species, and their relative abundances surpassed 95% in all five cultures. In contrast, other dominant bacteria at stage 0 decreased below 1% after enrichment. These results suggested that oils could promote the growth of Proteobacteria bacteria. Proteobacteria bacteria have been reported to have been associated with oil degradation. Todorova et al. (2014) suggested that Proteobacteria were the dominant species in marine sediments polluted by oil. Gao et al. (2015) reported that Proteobacteria bacteria were the key players in cultures enriched from deep-sea sediments from the South Mid-Atlantic Ridge, with crude oil as the sole source of carbon and energy. Oyetibo et al. (2021) revealed that Proteobacteria was the dominant species in the bacterial community of marine sediments under the effects of oil. In addition, many other studies have demonstrated that Proteobacteria are common hydrocarbon-utilizing bacteria during the biodegradation of oils in deep-sea environments (Hazen et al., 2010; Kostka et al., 2011; Gao et al., 2015).

**FIGURE 3:**
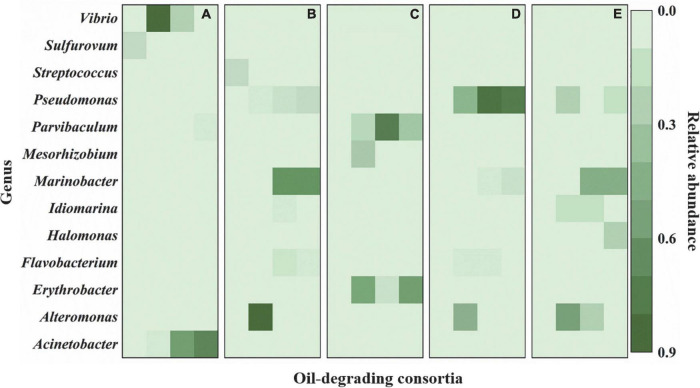
Variations in bacterial community compositions at genus level during enrichments. **(A)** The culture F derived from sediment F; **(B)** the culture J derived from sediment J; **(C)** the culture I derived from sediment I; **(D)** the culture N derived from sediment N; **(E)** the culture G derived from sediment G. In **(A–E)**, each column from left to right represent cultures before enrichment, after the first enrichment, after the second enrichment, and after the third enrichment, respectively. The green bar represents the relative abundance of species.

Similar to those at the phylum level, bacterial community compositions also exhibited variations at the genus level. Three kinds of change trends were observed for the relative abundance of the top 20 genera during the whole enrichment. The relative abundance of some bacteria exhibited decreasing trends during enrichment, where *Streptococcus* and *Sulfurovum* decreased to undetectable levels in cultures F and J. By contrast, other bacteria showed increasing trends during the entire enrichment. The low abundance of *Pseudomonas* and *Marinobacter* in cultures J and N, *Halomonas* in culture G, and *Acinetobacter* in culture F increased to be dominant genera after enrichment. In addition, some bacteria increased after the first enrichment. They dropped after the second and third enrichments, including *Mesorhizobium* in culture I, *Vibrio* in culture F, *Alteromonas* in cultures J, N, and G, *Idiomarina* in cultures J and G, and *Parvibaculum* and *Erythrobacter* in culture I (Figure 3 and Supplementary Figure 5).

### Characterization of oil-degrading consortia

In this study, bacterial community structures showed similarities and differences in five different oil-degrading consortia, which were enriched from the deep-sea sediments of the Haima cold seep areas (Figure 3 and Supplementary Figure 5). A total of 228 OTUs were identified among the five oil-degrading consortia. Consortium I comprised the most significant number of OTUs (140). For the other consortia, consortia F, J, N, and G contained only 60, 56, 73, and 62 OTUs, respectively (Supplementary Figure 6). These results indicated that species of oil-degrading bacteria might be correlated with geographic locations of sediments (Wang et al., 2014).

Proteobacteria was the most predominant of the five oil-degrading consortia at the phylum level. Their relative abundances were 98.3, 95.8, 96.6, 97.2, and 99.0% in consortia F, J, I, N, and G, respectively (Figure 2). However, at the genus level, the diversities and abundances of the dominant genera in oil-degrading consortia were distinct from each other. As depicted in Figure 3 and Supplementary Figure 5, the genera *Acinetobacter* and *Alcanivorax* were only markedly enriched in consortia F, accounting for their relative abundances of 74.5 and 5.1%, respectively. *Parvibaculum* (30.5%) and *Erythrobacter* (59.8%) were highly abundant in consortium I and distinguished from other consortia. The genera *Pseudomonas* and *Marinobacter* were two commonly abundant bacteria, with relative abundances of 14.9, 81.9, and 10.9% and 67.2, 9.7, and 51.9% in consortia J, N, and G, respectively. In contrast, *Idiomarina* and *Halomonas* were two other dominant genera in consortium G, and their relative abundances were 5.1 and 24.2%, respectively. These results revealed the selective effects of oils on the bacterial compositions of cultures derived from deep-sea sediments of the Haima cold seep areas, with genera of *Parvibaculum*, *Erythrobacter*, *Acinetobacter*, *Alcanivorax*, *Pseudomonas*, *Marinobacter*, *Halomonas*, and *Idiomarina* enriched as the dominant genera (relative abundance > 5%). Previous studies reported that some oil-degrading bacterial genera were present at low or undetectable levels before oil pollution, but they were found to predominate in oil-polluting marine environments (Brooijmans et al., 2009; Oyetibo et al., 2021).

**FIGURE 4:**
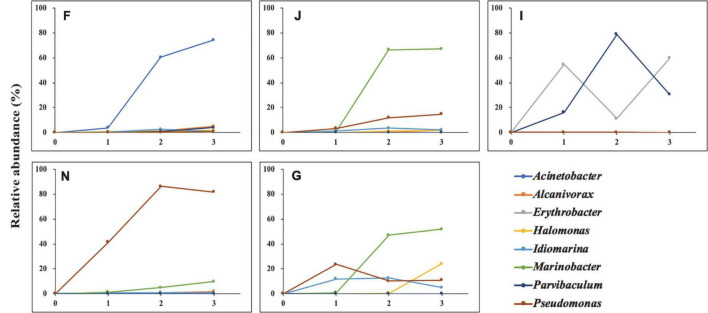
Variations in the relative abundance of the dominated genera of five oil-degrading consortia during the whole enrichments. 0, 1, 2, and 3 represent cultures before, after the first, after the second, and after the third enrichment. F, J, I, N, and G refer to five different cultures derived from five surface sediments. 100% is the total abundance of all species for each consortium.

Oils were provided as the sole carbon and energy sources for shaping oil-degrading consortia F, J, I, N, and G. Hence, the enriched genera in five oil-degrading consortia could be considered bacteria to be related to oil degradation. The variation in their abundances during enrichment is depicted in Figure 4. The abundance of the *Erythrobacter* dramatically increased after the first enrichment, decreased after the second enrichment, and finally increased to become dominant genera in consortium I, showing an inclined “*N*” curve. The genera of *Acinetobacter* in consortium F and *Marinobacter* in consortia J and G all showed markedly increasing trends during the whole enrichment period. In contrast, *Alcanivorax* in consortium F, *Halomonas* in consortium G, and *Marinobacter* in consortium N showed slight increases during the enrichments. The abundance of *Idiomarina* in consortium G and *Parvibaculum* in consortium I increased during the first and second enrichments and decreased after the third enrichment. The abundance of *Pseudomonas* exhibited two patterns: It continuously increased during the enrichments in consortia J and N and showed an inverted “V” curve in consortium G during the enrichments. These results indicated that species of oil-degrading bacteria might be correlated with geographic locations of sediments (Wang et al., 2014).

The dominant genera in the five oil-degrading consortia have all been correlated with oil degradation. For example, *Alcanivorax* and *Marinobacter* species are good marine hydrocarbon-degrading bacteria (Kasai et al., 2002; Dastgheib et al., 2011; Al-Mailem et al., 2013; Fathepure, 2014). In addition, *Parvibaculum* and *Idiomarina* bacteria have been identified as oil degraders in marine environments (Wang et al., 2010; Fakhrzadegan et al., 2019). Many species in the genus *Acinetobacter* have been successfully isolated from different environments and have shown the oil degradation ability for oils (Shiri et al., 2014; Acer et al., 2016, 2020; Nkem et al., 2016; Ma et al., 2021a; Shi et al., 2021). *Pseudomonas* bacteria were also reported to be capable of degrading oils (Xue et al., 2015; Varjani and Upasani, 2016; Gao et al., 2019). In addition, *Pseudomonas* bacteria were usually enriched as significant components after hydrocarbon pollution environments (Vandera and Koukkou, 2017). Strains in the genera *Erythrobacter* and *Halomonas* contributed to hydrocarbon degradation in marine environments (Röling et al., 2002; Gao et al., 2015; Gutierrez et al., 2015; Neifar et al., 2019; Perez Calderon et al., 2019; Peng et al., 2020).

### Isolation and identification of potential oil-degrading bacteria

**FIGURE 5:**
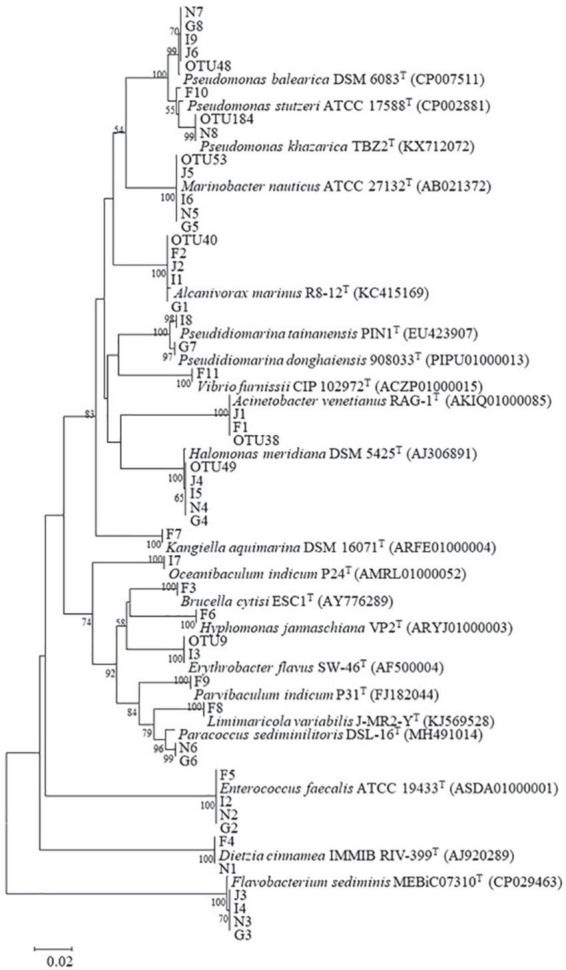
Neighbor-joining phylogenetic tree of representative OTU sequences, isolates, and their related species. Bootstrap values (expressed as percentages of 1,000 replications) greater than 50% are shown at branch points. Bar, 0.05 substitutions per nucleotide position. Accession numbers are given in parentheses.

Forty-two bacterial strains were obtained from five oil-degrading consortia and were affiliated with 18 genera belonging to Actinobacteria (2 strains), Bacteroidetes (4 strains), Firmicutes (4 strains), Alphaproteobacteria (8 strains), and Gammaproteobacteria (24 strains). Among them, Gammaproteobacteria was the largest class and comprised *Pseudidiomarina* (2 strains), *Marinobacter* (4 strains), *Alcanivorax* (4 strains), *Halomonas* (4 strains), *Kangiella* (1 strain), *Acinetobacter* (2 strains), *Pseudomonas* (6 strains), and *Vibrio* (1 strain). Alphaproteobacteria was the second class and was composed of *Brucella* (1 strain), *Parvibaculum* (1 strain), *Hyphomonas* (1 strain), *Limimaricola* (1 strain), *Paracoccus* (2 strains), *Oceanibaculum* (1 strain), and *Erythrobacter* (1 strain). In contrast, other phyla of Actinobacteria, Bacteroidetes and Firmicutes were only composed of *Dietzia* (2 strains), *Flavobacterium* (4 strains), and *Enterococcus* (4 strains) (Figure 5 and Table 1). Gammaproteobacteria dominated the bacterial communities of marine environments polluted by oils (Head et al., 2006; Yakimov et al., 2007; Kostka et al., 2011; Wang et al., 2014). In the Macondo well oil plume, Gammaproteobacteria was major hydrocarbon-oxidizing bacteria in microbial communities (Hazen et al., 2010). Therefore, it was reasonable that most of the strains were affiliated with Gammaproteobacteria.

**TABLE 1:** Taxonomic identification of bacterial isolates recovered from the five oil-degrading consortia of F, J, I, N, and G derived from five surface sediments.

Consortia	Isolate	Closest type strains	EzBioCloud accession number	Similarity (%)
	F1	*Acinetobacter venetianus* RAG-1	AKIQ01000085	99.86
	F2	*Alcanivorax marinus* R8-12	KC415169	99.86
	F3	*Brucella cytisi* ESC1	AY776289	100
	F4	*Dietzia cinnamea* IMMIB RIV-399	AJ920289	99.42
	F5	*Enterococcus faecalis* ATCC 19433	ASDA01000001	99.65
F	F6	*Hyphomonas jannaschiana* VP2	ARYJ01000003	99.77
	F7	*Kangiella aquimarina* DSM 16071	ARFE01000004	99.28
	F8	*Limimaricola variabilis* J-MR2-Y	KJ569528	99.92
	F9	*Parvibaculum indicum* P31	FJ182044	100
	F10	*Pseudomonas stutzeri* ATCC 17588	CP002881	99.43
	F11	*Vibrio furnissii* CIP 102972	ACZP01000015	99.86
	J1	*Acinetobacter venetianus* RAG-1	AKIQ01000085	99.93
	J2	*Alcanivorax marinus* R8-12	KC415169	99.93
	J3	*Flavobacterium sediminis* MEBiC07310	CP029463	99.71
	J4	*Halomonas meridiana* DSM 5425	AJ306891	99.93
J	J5	*Marinobacter nauticus* ATCC 27132	AB021372	99.43
	J6	*Pseudomonas balearica* DSM 6083	CP007511	99.57
	I1	*Alcanivorax marinus* R8-12	KC415169	99.79
	I2	*Enterococcus faecalis* ATCC 19433	ASDA01000001	99.65
	I3	*Erythrobacter flavus* SW-46	AF500004	99.71
	I4	*Flavobacterium sediminis* MEBiC07310	CP029463	99.71
I	I5	*Halomonas meridiana* DSM 5425	AJ306891	99.64
	I6	*Marinobacter nauticus* ATCC 27132	AB021372	99.64
	I7	*Oceanibaculum indicum* P24	AMRL01000052	99.85
	I8	*Pseudidiomarina tainanensis* PIN1	EU423907	99.93
	I9	*Pseudomonas balearica* DSM 6083	CP007511	99.79
	N1	*Dietzia cinnamea* IMMIB RIV-399	AJ920289	99.57
	N2	*Enterococcus faecalis* ATCC 19433	ASDA01000001	99.65
N	N3	*Flavobacterium sediminis* MEBiC07310	CP029463	99.28
	N4	*Halomonas meridiana* DSM 5425	AJ306891	99.79
	N5	*Marinobacter nauticus* ATCC 27132	AB021372	99.78
	N6	*Paracoccus sediminilitoris* DSL-16	MH491014	97.72
N	N7	*Pseudomonas balearica* DSM 6083	CP007511	99.43
	N8	*Pseudomonas khazarica* TBZ2	KX712072	99.36
	G1	*Alcanivorax marinus* R8-12	KC415169	99.86
	G2	*Enterococcus faecalis* ATCC 19433	ASDA01000001	99.58
	G3	*Flavobacterium sediminis* MEBiC07310	CP029463	99.78
G	G4	*Halomonas meridiana* DSM 5425	AJ306891	99.79
	G5	*Marinobacter nauticus* ATCC 27132	AB021372	99.64
	G6	*Paracoccus sediminilitoris* DSL-16	MH491014	97.36
	G7	*Pseudidiomarina donghaiensis* 908033	PIPU01000013	99.43
	G8	*Pseudomonas balearica* DSM 6083	CP007511	99.93

In this study, bacterial growth tests revealed that 21 potential oil-degrading isolates exhibited vigorous growth with 1 g/L oil (the ratio of crude oil to diesel oil = 1:1) as the sole carbon and energy source. They were closely related to *Acinetobacter venetianus* (strains F1 and J1), *Alcanivorax marinus* (strains F2, J2, I1, and G1), *Dietzia cinnamea* (strains F4 and N1), *Enterococcus faecalis* (strain G2), *Flavobacterium sediminis* (strains I4, N3, and G3), *Halomonas meridiana* (strains I5 and N4), *Kangiella aquimarina* (strain F7), *Limimaricola variabilis* (strain F8), *Marinobacter nauticus* (strains J5 and N5), *Paracoccus sediminilitoris* (strain N6), *Pseudomonas khazarica* (strain N8), and *Vibrio furnissii* (strain F11). Although other strains were successfully isolated from oil-degrading consortia, they did not display good growth on oils. This was probably because some bioavailable metabolites were produced during the degradation of oils by degrading consortia and supported the growth of these strains (Vandera and Koukkou, 2017; Zhang et al., 2020).

Twenty-one potential oil-degrading isolates have been partly verified to be oil degraders in marine environments. Strains of *Alcanivorax marinus* and *Pseudomonas khazarica* were successfully isolated and identified as oil-degrading bacteria from marine sediments (Lai et al., 2013; Tarhriz et al., 2020; Jagtap et al., 2021). In addition, many other members belonging to these two genera were isolated from deep-sea sediments or seawater (Kimata et al., 2004; Liu and Shao, 2005; Lai et al., 2013, 2016; Gao et al., 2015; Yang et al., 2018; Dong et al., 2021). Strains of *Acinetobacter venetianus* and *Marinobacter nauticus* were not obtained from marine environments, and their oil degradation ability was also not reported in other environments in previous studies. However, numerous strains in these two genera were widely distributed in marine environments (Gao et al., 2013; Mahjoubi et al., 2013; Cui et al., 2016) and were characterized by their capability to degrade hydrocarbons (Di Cello et al., 1997; Luckarift et al., 2011; Lee et al., 2012; Fondi et al., 2016; Bendadeche et al., 2019; Fakhrzadegan et al., 2019). Although *Dietzia cinnamea* and *Enterococcus faecalis* strains were not obtained from marine environments, they were isolated from other environments and showed degradation ability for petroleum hydrocarbons (von der Weid et al., 2007; Bihari et al., 2011; Boontawan and Boontawan, 2011; Procópio et al., 2013; Vignaroli et al., 2013; Chen et al., 2017; Dilmi et al., 2017). Strains of *Flavobacterium sediminis*, *Halomonas meridiana*, *Kangiella aquimarina*, *Vibrio furnissii*, and *Paracoccus sediminilitoris* were isolated from marine sediments (James et al., 1990; Yoon et al., 2004; Hassanshahian, 2014; Bae et al., 2018; Wei et al., 2019), but there was no report of their ability to degrade oils. In contrast, other bacteria of these genera from marine environments showed their hydrocarbon degradation ability, including *Flavobacterium* (*Flavobacterium petrolei* sp. nov., *Flavobacterium naphthae* sp. nov., and *Flavobacterium beibuense* sp. nov.) (Fu et al., 2011; Chaudhary and Kim, 2018; Chaudhary et al., 2019), *Halomonas* (*Halomonas profundus* sp. nov., *Halomonas* sp. strain BS53, and *Halomonas lionensis* sp. nov.) (Simon-Colin et al., 2008; Gaboyer et al., 2014; Fakhrzadegan et al., 2019), *Kangiella* (*Kangiella profundi* sp. nov., *Kangiella geojedonensis* sp. nov., and *Kangiella* sp. strain DP40) (Romanenko et al., 2010; Yoon et al., 2012; Li et al., 2015; Fakhrzadegan et al., 2019), and *Vibrio* (*Vibrio* sp. strain NW4, and *Vibrio* sp. strain DS35) (Fakhrzadegan et al., 2019). *Limimaricola variabilis* species have not been isolated from natural environments in previous studies. However, other strains in the genus *Limimaricola* were isolated from marine environments. There was also no report of oil degradation ability by bacteria in this genus (Wang et al., 2015).

### Degradation of alkanes by high-efficiency oil-degrading strains

According to bacterial growth ability on oils, different bacterial genera, and visual observations, seven strains of F1, F2, F7, F8, J5, N3, and N6 were selected from 21 potential oil-degrading strains for further biodegradation tests of alkanes (Supplementary Figure 7). The degradation rates of alkanes (C_10_–C_35_) were calculated following the equation in section “Alkane degradation ability by high-efficiency oil-degrading strains.” In control groups, the average weight loss of C_10_–C_15_, C_16_–C_20_, C_21_–C_25_, C_26_–C_30_, C_31_–C_35_, and C_10_–C_35_ in crude oil was 33.2, 4.6, 8.1, 5.6, 3.7, and 57.7 μg, respectively. Correspondingly, they were 254.7, 80.0, 14.7, 1.0, 0.0, and 350.4 μg in diesel oil. The tested isolates exhibited high degradation efficiency for total alkanes, ranging from 70.3 to 78.0% and 62.7 to 66.3% in crude and diesel oil, respectively (Supplementary Table 1). Moreover, alkane degradation efficiencies by seven isolates were all higher in crude oil than in diesel oil, which was ascribed to the different components and contents of alkanes in crude oil and diesel oil at the beginning of incubation. The total content of alkanes was three times lower in crude oil (322.2 μg) than in diesel oil (1086.4 μg). The dominant alkane components were C_10_–C_15_, C_16_–C_20_, and C_21_–C_25_ in diesel oil, while alkanes were distributed evenly in crude oil. The contents of the alkanes of C10–C15, C16–C20, C21–C25, C26–C30, and C31–C35 were 65.6, 81.5, 89.1, 64.5, and 21.4 μg in crude oil, respectively. Correspondingly, their contents were 482.9, 470.2, 128.0, 5.3, and 0.03 μg in diesel oil, respectively (Supplementary Figure 8). Alkanes are proven toxic to microorganisms by changing cell membrane function and inhibiting cell growth (Sikkema et al., 1995; Singh et al., 2012; Chen et al., 2013; Kang and Nielsen, 2017). Therefore, it was reasonable that higher concentrations of alkanes caused lower degradation rates in diesel oil than in crude oil.

Among these seven strains, *Alcanivorax marinus* strain F2 and *Kangiella aquimarina* F7 showed the highest degradation rates for total alkanes of 78.0 ± 2.1% in crude oil and 66.3 ± 0.3% in diesel oil, respectively. They showed the degradation rates for total alkanes of 65.5% in diesel oil and 72.5% in crude oil, respectively. As reported, *Alcanivorax* strains became predominant taxa after crude oil spills and played essential roles in the bioremediation of oil spills worldwide (Kasai et al., 2002; Hara et al., 2003; Harayama et al., 2004; McKew et al., 2007a,b; Yakimov et al., 2007, 2019; Rojo, 2009). In addition, other species of *Alcanivorax* were reported to utilize alkanes of all lengths (C_5_–C_36_) (Liu and Shao, 2005; Singh et al., 2012; Xia et al., 2019). Moreover, genes involved in alkane degradation, including cytochrome P450s, alkane monooxygenases, and monooxygenase, were all identified in *Alcanivorax* sp. strains (Hara et al., 2004; Throne-Holst et al., 2007; Wang and Shao, 2013; Xia et al., 2019; Freitas et al., 2020; Zadjelovic et al., 2020). *Kangiella* sp. had also been reported to have relationships with hydrocarbon degradation, but there was no report of their degradation ability for alkanes (Fakhrzadegan et al., 2019; Freitas et al., 2020).

The other five bacteria also showed remarkable degradation rates but small differences in their ability to degrade total alkanes. *Acinetobacter venetianus* strain F1, *Limimaricola variabilis* strain F8, *Marinobacter nauticus* strain J5, *Flavobacterium sediminis* N3, and *Paracoccus* sp. strain N6 had degradation rates for total alkanes (C_10_–C_35_) of 74.9, 76.0, 70.3, 74.6, and 73.4% in crude oil and 62.7, 66.0, 64.0, 65.8, and 65.4% in diesel oil, respectively (Supplementary Table 1). The current findings were partly consistent with results from previous studies. Specifically, *Acinetobacter* sp. strain was found capable of utilizing alkanes of chain length C_10_–C_40_ in marine environments (Singh et al., 2012; Bendadeche et al., 2019). *Marinobacter* bacteria had a high degradation ability for short-chain alkanes of C_8_–C_10_, and no degradation was observed for long-chain alkanes of C_15_–C_23_ (Striebich et al., 2014). Marine strains in the genera *Flavobacteria* and *Paracoccus* were able to degrade alkanes. For example, *Flavobacterium* sp. DS-71 isolated from deep-sea sediments could utilize alkanes of chain length < C_25_ (Moriya and Horikoshi, 2002; Guibert et al., 2016). *Paracoccus* sp. strains were reported to utilize *n*-alkanes (Zhang et al., 2004). In contrast, there have been no reports about the degradation ability of *Limimaricola* sp. strains for alkanes.

**FIGURE 6:**
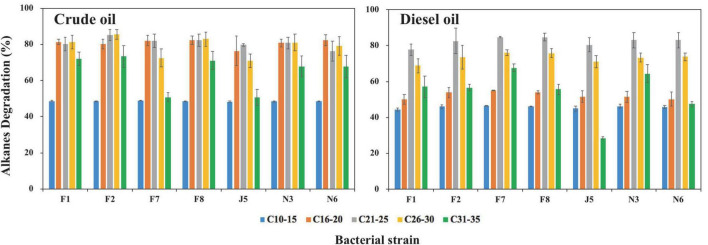
Degradation of alkanes in crude oil and diesel oil by seven oil-degrading strains after 20-day incubation in MMC medium. Error bars represent standard deviations of means (*n* = 3). F1, F2, F7, F8, J5, N3, and N6 refer to strain *Acinetobacter venetianus* F1, *Alcanivorax marinus* F2, *Kangiella aquimarina* F7, *Limimaricola variabilis* F8, *Marinobacter nauticus* J5, *Flavobacterium sediminis* N3, and *Paracoccus sediminilitoris* N6, respectively.

We also analyzed the degradation rates of alkanes with different carbons in crude oil and diesel oil (Figure 6). The degradation rates of seven strains for alkanes were all medium length > long chains > short chains. In crude oil, the degradation rates were C_21_–C_30_ > C_31_–C_35_ > C_10_–C_15_, while the degradation rates were C_21_–C_30_ > C_31_–C_35_ > C_10_–C_20_ in diesel oil. Specifically, degradation rates by seven strains for C_16_–C_30_ ranged from 70.8 to 85.5% in crude oil, while there were only approximately 48.3–48.8% and 50.7–73.3% for C_10_–C_15_ and C_31_–C_35_, respectively. In contrast, the degradation rates of C_21_–C_30_, C_31_–C_35_, and C_10_–C_20_ ranged from 68.8 to 84.7%, from 28.4 to 67.7%, and from 44.4 to 55.1% in diesel oil, respectively. The degradation rates of short-chain alkanes were worse than those of long-chain alkanes because short-chain alkanes are usually toxic to bacteria, and long-chain alkanes have low solubility and bioavailability (Hassanshahian et al., 2014; Fuentes et al., 2015; Guermouche M’rassi et al., 2015; Vandera and Koukkou, 2017). The degradation rates of medium-chain alkanes were faster than those of long-chain alkanes, which was due to the higher hydrophobicity of long-chain alkanes (Smits et al., 2002; Throne-Holst et al., 2006; Feng et al., 2007; Sanscartier et al., 2009; Singh et al., 2012; Liu et al., 2014). Unlike our results, Shi et al. (2021) reported that the degradation rates of short-chain alkanes of C_10_–C_19_ were higher than those of long-chain alkanes of C_31_–C_35_. Ma et al. (2021b) found that strains showed higher degradation rates for short-chain alkanes (C_10_–C_19_) and medium-chain alkanes (C_20_–C_24_) than long-chain alkanes (C_25_–C_34_). This difference was attributed to different bacterial species.

In summary, *Kangiella aquimarina*, *Acinetobacter venetianus*, *Limimaricola variabilis*, *Marinobacter nauticus*, *Flavobacterium sediminis*, and *Paracoccus sediminilitoris* were identified as oil-degrading bacteria from deep-sea environments for the first time. This study contributes to our understanding of marine oil-degrading bacterial diversity and provides a basis for in-depth research on the degradation mechanism of hydrocarbons and the deep-sea adaptability of microorganisms. Moreover, our study offers microbial resources for developing bioremediation technology for marine oil pollution and further studying the ecological environment significance of marine oil-degrading bacteria.

## Conclusion

In this study, five oil-degrading consortia were obtained from deep-sea sediments of the Haima cold seep, the South China Sea, with oil as the sole carbon source. Oils decreased bacterial community diversity and enriched *Parvibaculum*, *Erythrobacter*, *Acinetobacter*, *Alcanivorax*, *Pseudomonas*, *Marinobacter*, *Halomonas*, and *Idiomarina* as the dominant genera. We successfully isolated 42 strains from oil-degrading consortia. After degradation ability tests, seven oil-degrading strains (*Acinetobacter venetianus* strain F1, *Alcanivorax marinus* strain F2, *Kangiella aquimarina* strain F7, *Limimaricola variabilis* strain F8, *Marinobacter nauticus* strain J5, *Flavobacterium sediminis* strain N3, and *Paracoccus sediminilitoris* strain N6) were identified as high-efficiency degrading bacteria, with higher degradation rates in crude oil than in diesel oil. The degradation rates for alkanes were medium chains > long chains > short chains. This study is the first report about oil-degrading bacteria in the Haima cold seep areas, the South China Sea, which helps understand the oil-degrading bacterial biodiversity and expands degrading bacterial resources for oil bioremediation applications.

## Data availability statement

The datasets presented in this study can be found in online repositories. The names of the repository/repositories and accession number(s) can be found below: NCBI – PRJNA852871.

## Author contributions

LL: conceptualization, methodology, experiment, roles/writing—original draft, data curation, funding acquisition, and writing—review and editing. JL: methodology and data curation. YC and QL: investigation. ZM and LW: data curation. SZ: writing—review and editing, supervision, funding acquisition, and resources. All authors contributed to the article and approved the submitted version.
