# Supplementary material for: Degradation potential of alkanes by diverse oil-degrading bacteria from deep-sea sediments of Haima cold seep areas, South China Sea
Source: Front Microbiol. 2022 Oct 19;13:920067. doi: 10.3389/fmicb.2022.920067 (PMC9626528; doi:10.3389/fmicb.2022.920067)
Supplement: Supplementary file 1 [file Data_Sheet_1.docx]

**Supporting Material for:**

**Degradation potential of alkanes by diverse oil‑degrading bacteria from deep-sea sediments of Haima cold seep areas, South China Sea**

**Lina Lyu^1,*^, Jie Li^1,2^, Yu Chen^2^, Zhimao Mai^1^, Lin Wang^1^, Qiqi Li^1^, Si Zhang^1,2,*^**

^1^ CAS Key Laboratory of Tropical Marine Bio-resources and Ecology, South China Sea Institute of Oceanology, Chinese Academy of Sciences, Guangzhou 510301, Guangdong, China

^2^ Southern Marine Science and Engineering Guangdong Laboratory (Guangzhou), Guangzhou 511458, Guangdong, China

*Corresponding author:

CAS Key Laboratory of Tropical Marine Bio-resources and Ecology

South China Sea Institute of Oceanology, Chinese Academy of Sciences

164 Xingang Road, Guangzhou 510301, China.

E-mail address: zhsmid@scsio.ac.cn (S. Zhang); lvlina@scsio.ac.cn (L. Lyu)

Tel: +86-020-89023105

**Number of figures: 8**

**Number of tables: 1**


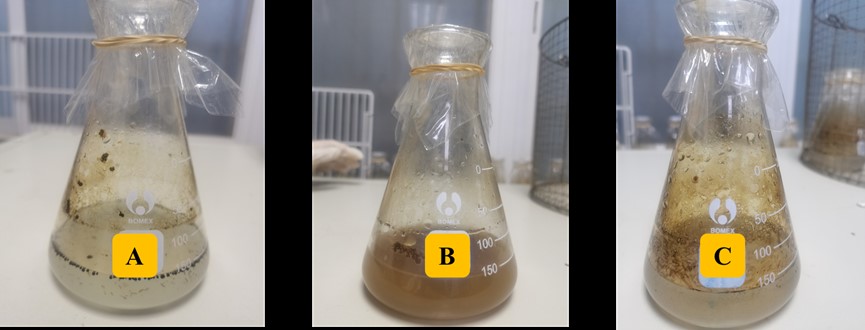


**Fig. S1** Three phenomena of bacterial cultures after bacterial growth test as described in the “2.6 Screening high-efficiency oil-degrading strains” section.


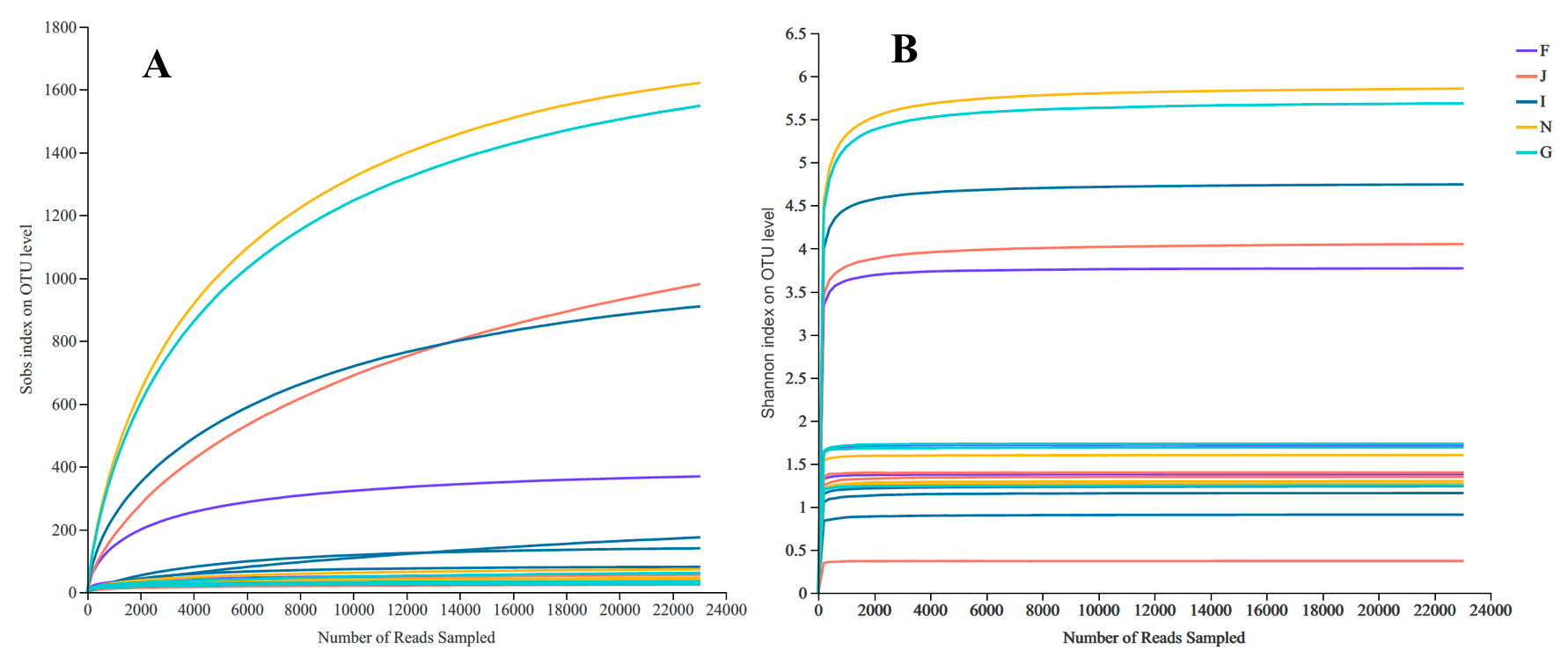


**Fig. S2** Rarefaction curves of bacterial communities shown by Sobs (A) and Shannon (B) index at the sequencing depth of 23037.

**
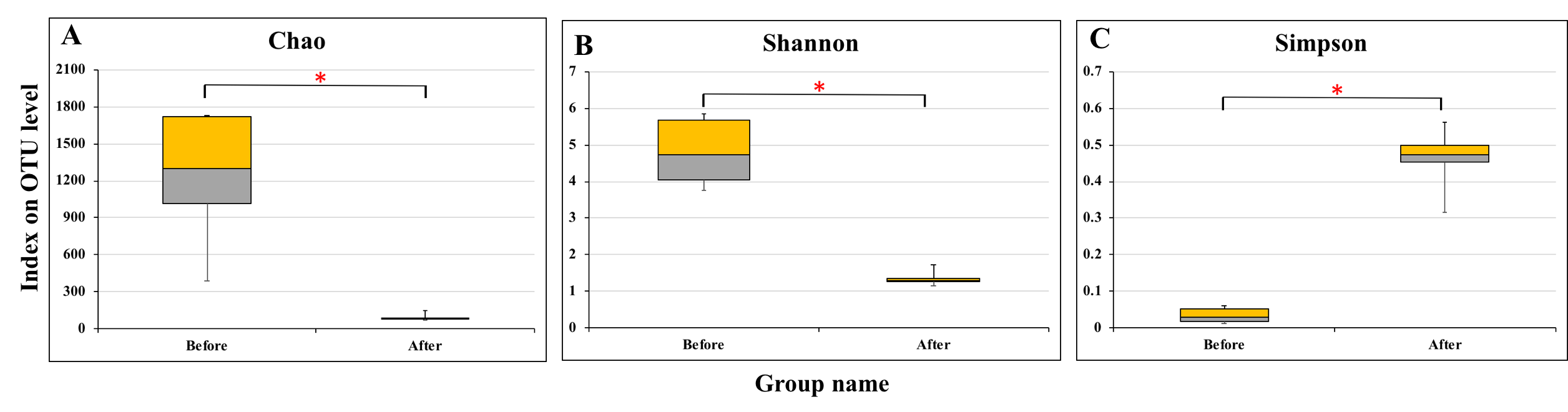
**

**Fig. S3** Wilcoxon rank-sum test for Chao (A), Shannon (B) and Simpson (C) index showing the significant differences between two groups of cultures before and after enrichments.


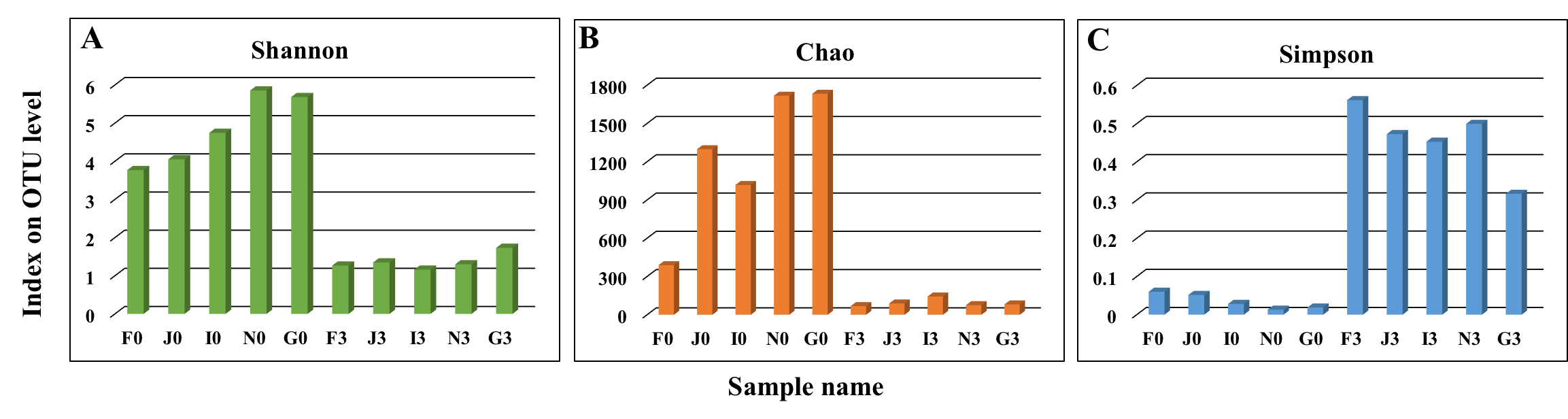


**Fig. S4** Alpha-diversity of bacterial community of cultures before enrichments (F0, J0, I0, N0, G0) and after enrichments (F3, J3, I3, N3, G3) . F, J, I, N and G represent five different cultures derived from five surface sediments.


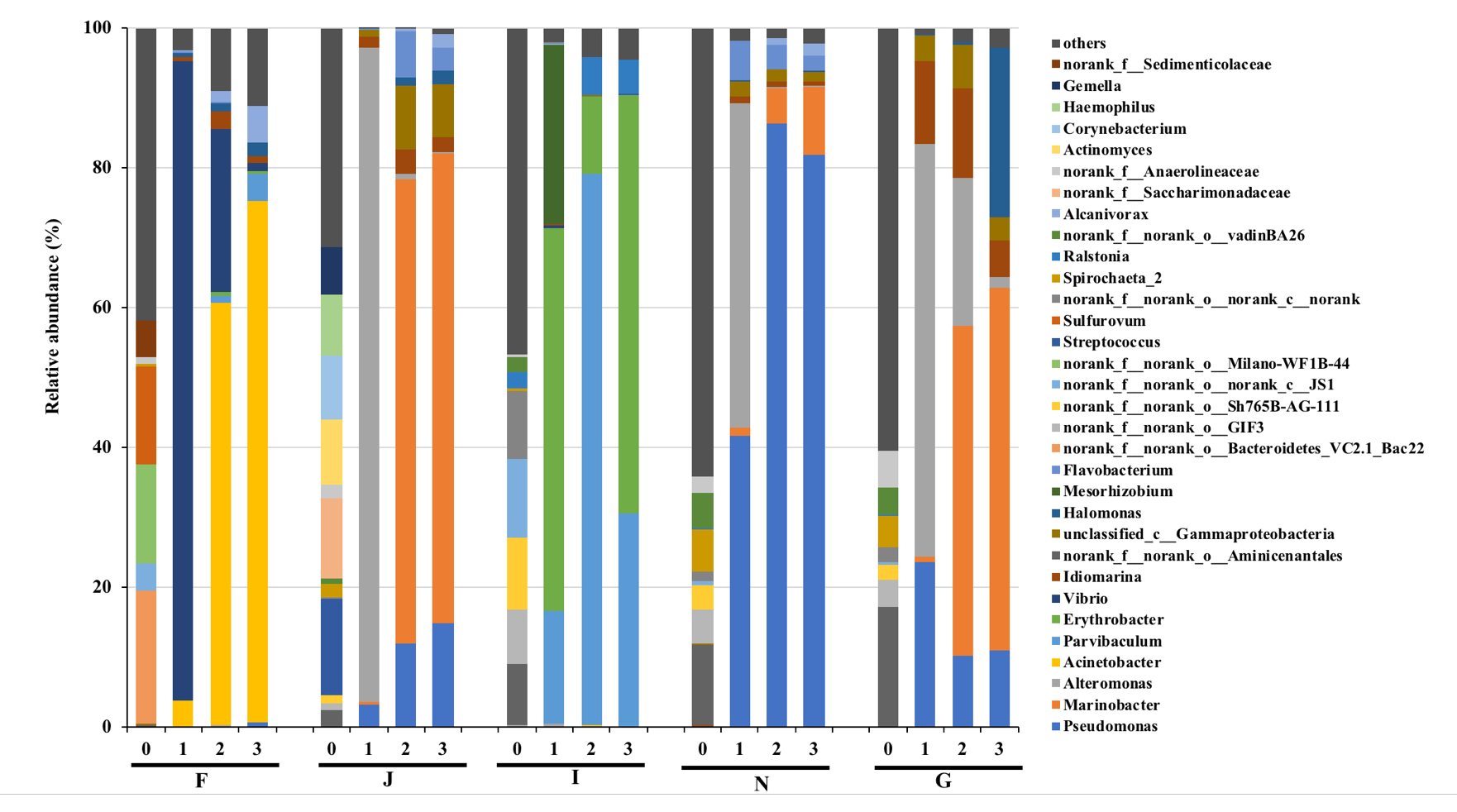


**Fig. S5** Variations in bacterial community compositions at genus level during enrichments. Stage 0, 1, 2, 3 represent cultures before enrichment, after the first enrichment, after the second enrichment and after the third enrichment, respectively. F, J, I, N, G refer to five different cultures derived from five surface sediments, respectively.


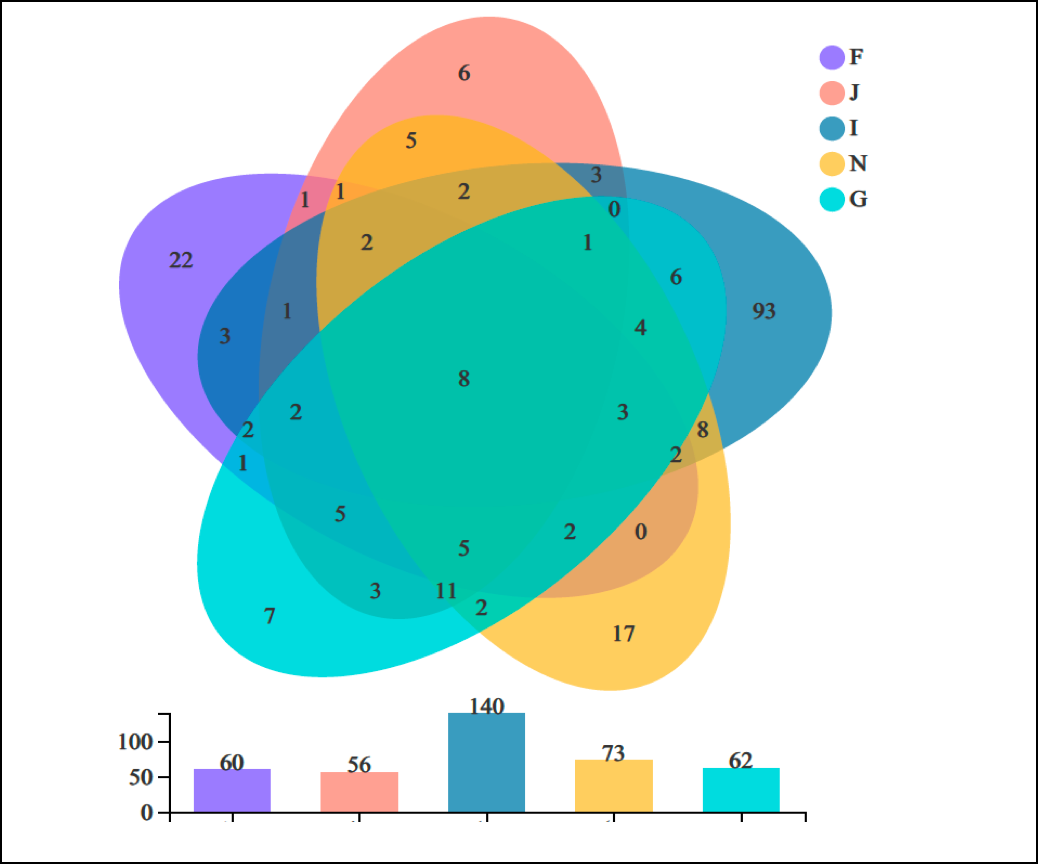


**Fig. S6** Venn diagram showing the shared and unique numbers of OTUs among five oil-degrading consortia of F, J, I, N, G.


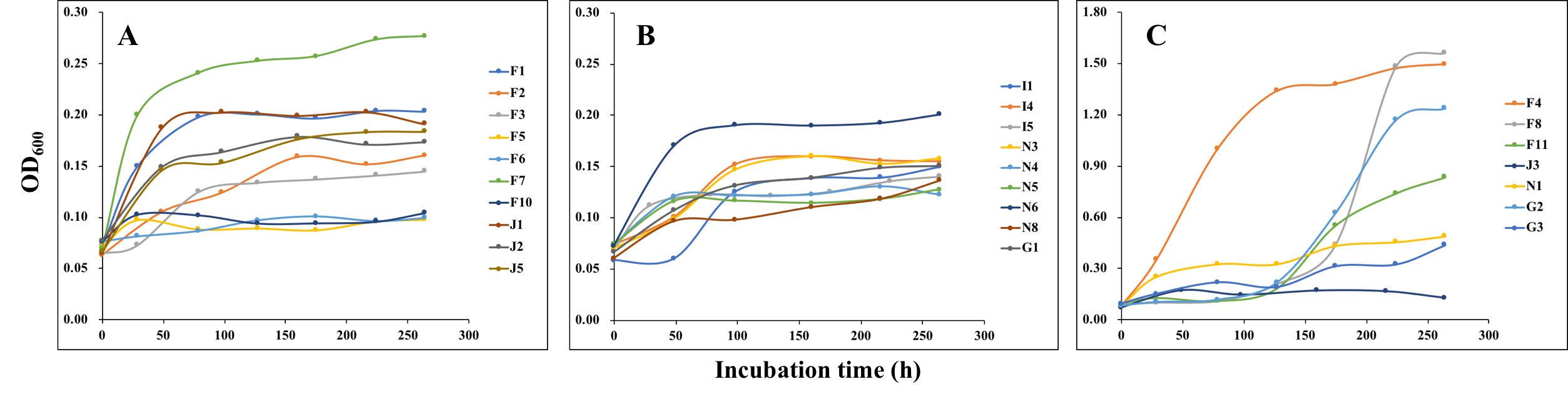


**Fig. S7** Bacterial growth curves in MMC medium with oils as the sole carbon source. X-axis, incubation time (hours), Y-axis, OD_600_ values.

**
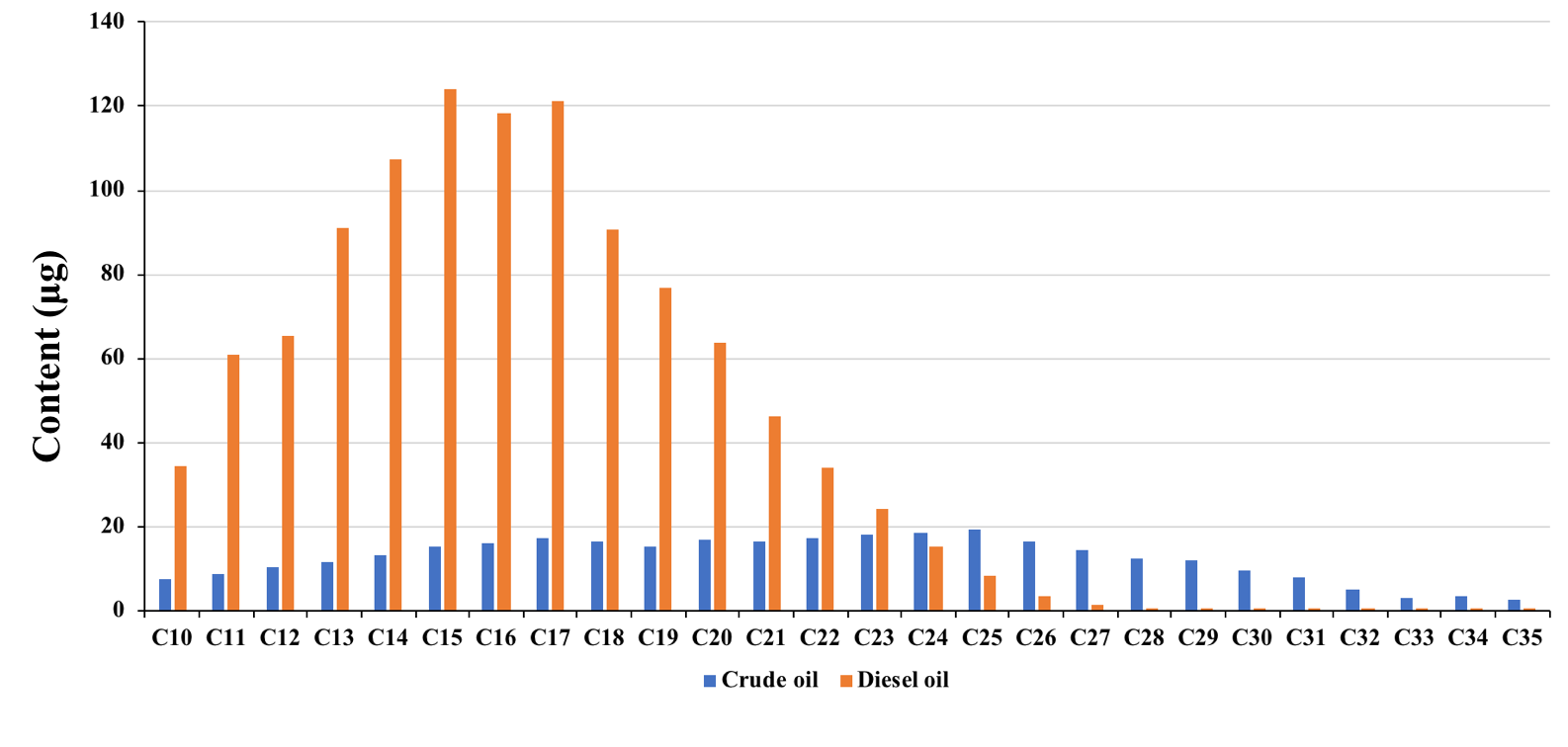
**

**Fig. S8** The contents of various alkanes in crude oil (A) and diesel oil (B) before incubation.

**Table S1** Average degradation rates (%) of alkanes in crude oil and diesel oil by oil-degrading strains after 20 days incubation in MMC medium. F1, F2, F7, F8, J5, N3 and N6 represent strain *Acinetobacter venetianus* F1, *Alcanivorax marinus* F2, *Kangiella aquimarina* F7, *Limimaricola variabilis* F8, *Marinobacter nauticus* J5, *Flavobacterium sediminis* N3 and *Paracoccus sediminilitoris* N6 , respectively.

| **Crude oil** | **F1** | **F2** | **F7** | **F8** | **J5** | **N3** | **N6** |
| --- | --- | --- | --- | --- | --- | --- | --- |
| C_10_-C_15_ | 48.6 | 48.7 | 48.8 | 48.7 | 48.3 | 48.4 | 48.6 |
| C_16_-C_20_ | 81.4 | 80.2 | 82.1 | 82.2 | 76.4 | 80.8 | 82.4 |
| C_21_-C_25_ | 80.2 | 85.1 | 81.9 | 82.2 | 79.7 | 80.8 | 76.4 |
| C_26_-C_30_ | 81.3 | 85.5 | 72.4 | 82.9 | 70.8 | 81.0 | 79.3 |
| C_31_-C_35_ | 71.9 | 73.3 | 50.8 | 71.1 | 50.7 | 67.9 | 67.7 |
| C_10_-C_35_ | 74.9 | 78.0 | 72.5 | 76.0 | 70.3 | 74.6 | 73.4 |
| **Diesel oil** | **F1** | **F2** | **F7** | **F8** | **J5** | **N3** | **N6** |
| C_10_-C_15_ | 44.4 | 46.0 | 46.5 | 46.2 | 44.9 | 46.2 | 45.8 |
| C_16_-C_20_ | 50.2 | 53.9 | 55.1 | 54.1 | 51.6 | 51.3 | 50.2 |
| C_21_-C_25_ | 77.7 | 82.6 | 84.7 | 84.7 | 80.4 | 83.0 | 83.1 |
| C_26_-C_30_ | 68.8 | 73.5 | 76.1 | 75.8 | 70.9 | 73.3 | 73.9 |
| C_31_-C_35_ | 57.1 | 56.6 | 67.7 | 55.7 | 28.4 | 64.4 | 47.5 |
| C_10_-C_35_ | 62.7 | 65.5 | 66.3 | 66.0 | 64.0 | 65.8 | 65.4 |
